# Cytochalasin B Treatment and Osmotic Pressure Enhance the Production of Extracellular Vesicles (EVs) with Improved Drug Loading Capacity

**DOI:** 10.3390/nano12010003

**Published:** 2021-12-21

**Authors:** Ashita Nair, Jiyoon Bu, Piper A. Rawding, Steven C. Do, Hangpeng Li, Seungpyo Hong

**Affiliations:** 1 Division of Pharmaceutical Sciences, School of Pharmacy, University of Wisconsin, Madison, WI 53705, USA; anair23@wisc.edu (A.N.); bu6@wisc.edu (J.B.); rawding@wisc.edu (P.A.R.); scdo@wisc.edu (S.C.D.); hli578@wisc.edu (H.L.); 2 Wisconsin Center for NanoBioSystems (WisCNano), School of Pharmacy, The University of Wisconsin-Madison, 777 Highland Ave., Madison, WI 53705, USA; 3 Department of Biomedical Engineering, College of Engineering, University of Wisconsin, Madison, WI 53706, USA; 4 Yonsei Frontier Lab, Department of Pharmacy, Yonsei University, Seoul 03722, Korea

**Keywords:** extracellular vesicles, membrane vesicles, exosome mimetic, cytochalasin B (CB), hypo-osmotic pressure, drug delivery

## Abstract

Extracellular vesicles (EVs) have been highlighted as novel drug carriers due to their unique structural properties and intrinsic features, including high stability, biocompatibility, and cell-targeting properties. Although many efforts have been made to harness these features to develop a clinically effective EV-based therapeutic system, the clinical translation of EV-based nano-drugs is hindered by their low yield and loading capacity. Herein, we present an engineering strategy that enables upscaled EV production with increased loading capacity through the secretion of EVs from cells via cytochalasin-B (CB) treatment and reduction of EV intravesicular contents through hypo-osmotic stimulation. CB (10 µg/mL) promotes cells to extrude EVs, producing ~three-fold more particles than through natural EV secretion. When CB is induced in hypotonic conditions (223 mOsm/kg), the produced EVs (hypo-CIMVs) exhibit ~68% less intravesicular protein, giving 3.4-fold enhanced drug loading capacity compared to naturally secreted EVs. By loading doxorubicin (DOX) into hypo-CIMVs, we found that hypo-CIMVs efficiently deliver their drug cargos to their target and induce up to ~1.5-fold more cell death than the free DOX. Thus, our EV engineering offers the potential for leveraging EVs as an effective drug delivery vehicle for cancer treatment.

## 1. Introduction

Extracellular vesicles (EVs) are naturally occurring, cell-secreted nanoparticles composed of a lipid bilayer membrane with embedded transmembrane proteins (i.e., cell surface receptors) which encloses a cytoplasm that contains biological information of parental cells (i.e., proteins, DNA, and RNA) [1,2,3,4,5]. EVs function as messengers of intracellular communication by protecting their cellular contents while simultaneously delivering information to specific recipient cells [1]. Many efforts have been made to take advantage of the intrinsic messaging abilities of EVs and utilize them as drug delivery carriers. Unlike synthetic nanoparticles, this natural drug carrier is stable under various physiological and pathological conditions and has a less immunogenic and cytotoxic profile [6]. Owing to their unique properties, EVs have been leveraged to deliver RNA [7], enzymes [8], and various chemotherapeutics [9,10].

Although these achievements have garnered remarkable scientific and clinical attention, significant barriers limit their clinical translation of EV-based drug delivery systems. Two of the biggest challenges include the upscaling of EV production and the enhancement of drug loading capacity [11,12]. To increase EV production, vesicles have been artificially produced by mechanically extruding or lysing donor cells (i.e., freeze-thaw, electroporation, and sonication) [11]. Despite the profound success in generating a sufficient number of membrane vesicles, these methods have been reported to induce chemical/physical damage on membrane lipid bilayer proteins, alter the surface zeta potential, exhibit size difference with naturally secreted EVs, and form large aggregates [8,13,14,15,16,17]. The artificially engineered membrane vesicles thus exhibit different biophysical properties (i.e., size and molecular contents) to those of naturally released EVs, and have reduced drug loading capacity, increased cytotoxic effects, and decreased delivery efficiency [15,16,17,18]. Alternatively, various modulators have been exploited to increase EV secretion from cells. Numerous studies have affirmed that hypoxia, or cellular proteins released as a response to hypoxia (i.e., hypoxia-inducible factor 1; HIF-1), promote angiogenesis by accelerating the secretion of EVs that facilitate tumor intercellular communication [19,20]. However, the observed increase in secretion was insignificant (~1.3-fold), limiting the utilization of hypoxia-induced EVs as drug delivery vehicles [20].

Additional efforts for upscaled EV production have been focused on the utilization of cytochalasin B (CB) as a mediator [21,22]. This cell-permeable mycotoxin is reported to block actin polymerization, and promote cells to extrude membrane vesicles throughout the extracellular domain [21]. The CB treatment has thus been demonstrated to produce a large number of vesicles from the cell membrane, facilitating the efficient scale-up of EV production. Recent studies have demonstrated that these CB-induced membrane vesicles (CIMVs) exhibit similar biophysical properties to their parental cells, as well as naturally secreted EVs [21]. CIMVs are thus being employed in therapeutics (i.e., immunomodulators using CIMVs generated from mesenchymal stem cells) and in various biomedical applications, including olfactory biosensors and cancer diagnostic systems [21,22,23,24]. 

In this study, we utilized CB to generate EVs from cell membrane and investigated their potential as drug delivery vehicles. In addition to the CB treatment, we induced hypo-osmotic pressure during EV production to enhance their loading capacity, which is another big challenge of the EV-based drug delivery systems. Previous studies have shown that under hypotonic conditions, cells release their intracellular contents to the exterior domain. We utilized this phenomenon, along with CB treatment, to accelerate EV production and reduce intravesicular content for the efficient loading and delivery of chemo-drugs. The effect of CB treatment and/or hypo-osmotic pressure on EV production and the biophysical properties of EVs were investigated, including the size, morphology, surface charge, cellular uptake, and amount of intravesicular proteins. The enhanced drug loading capacity of CIMVs obtained under hypotonic conditions (hypo-CIMVs) was confirmed using differently charged generation 7 (G7) poly(amidoamine) (PAMAM) dendrimers as model drugs. Doxorubicin hydrochloride (DOX), one of the most common chemotherapeutic drugs used to treat of a broad spectrum of tumors [25], was then loaded into the hypo-CIMVs to increase its delivery to tumor cells. We compared the cytotoxicity of DOX-loaded hypo-CIMVs with free DOX using in vitro assay to evaluate the payload delivery efficiency. This study provides design cues to efficiently upscale EV production and increase the delivery efficacy of chemo-drugs, resulting in promising directions for enhanced chemotherapy delivery.

## 2. Materials and Methods

### 2.1. Materials

Polyamidoamine (PAMAM) dendrimers with an ethylenediamine core of generation 7 were obtained from Dendritech Inc. (Midland, MI, USA). Cytochalasin B was purchased from Tocris, Bio-Techne Corporation (Minneapolis, MN, USA). Bovine serum albumin (BSA) was obtained from Millipore Sigma (St. Louis, MO, USA). NHS-Rhodamine mixed isomer (5/6-carboxy-tetramethyl-rhodamine succinimidyl ester, DiO cell-labeling solution (DiO), and bicinchoninic acid assay (BCA) kit were purchased from Thermo Fisher Scientific Inc. (Waltham, MA, USA). Cell Counting Kit-8 Assay (CCK8) for cell viability testing was purchased from Enzo Life Sciences, Inc. (Farmingdale, NY, USA). DMEM Media was obtained from Corning Incorporated (Corning, NY, USA). All other chemicals were obtained from Sigma-Aldrich (St. Louis, MO, USA).

### 2.2. EV Production and Isolation

786-O and ACHN cells were cultured as a monolayer at 80% confluence at 37 °C, with 5% CO_2_ in DMEM media supplemented with 1% penicillin/streptomycin antibiotics and 10% heat-inactivated fetal bovine serum (FBS). The EVs for all experiments were isolated from the conditioned cell culture media of cultured 786-O and ACHN. To generate the conditioned media, the monolayer cells were incubated in their respective media supplemented with antibiotics and 1% BSA for 24 h. Note that the media was filtered with 0.22 µm Steriflip-GP filters (Millipore) prior to the cell incubation. The exosome isolation process was carried out using the ultracentrifugation method [26]. Briefly, collected cell culture media was centrifuged at 300× *g* for 10 min to collect dead cells, followed by centrifugation at 4000× *g* for 30 min to collect any large cellular debris. The media was then centrifuged at 100,000× *g* for 1 h using a Beckman Type 45 Ti rotor and ultracentrifuge. The collected pellets were then washed with PBS and recentrifuged to remove any remaining media. The pelleted exosomes were resuspended in PBS and stored at −80 °C until further use. For hypotonic conditions, the complete cell media was mixed with water supplemented with D-glucose (25 mM), sodium bicarbonate (44 mM), and sodium monophosphate (0.91 mM) at different ratios to achieve the desired osmolality. To collect EVs generated by cells in different conditions, the cells were incubated in modified hypotonic media supplemented with 1% BSA.

### 2.3. Dendrimer Preparation, Characterization, and Modification

Dendrimers were conjugated to NHS-Rhodamine and then surface-modified, following methods previously published [27]. Briefly, PAMAM dendrimers (G7-NH2; 0.26 × 10^−6^ mol) were dissolved in phosphate-buffered saline at pH 7.6. NHS-Rhodamine (2.1 × 10^–6^ mol) was dissolved in DMSO and was added dropwise to the dendrimer with constant stirring. The mixture was reacted for 24 h at room temperature and then purified using a Spectra/Por dialysis membrane with MWCO of 10,000 Da (Spectrum Laboratories Inc., Rancho Dominguez, CA, USA) against water for purification. The purified dendrimers were then freeze-dried using a Labconco FreeZone 4.5 system (Kansas City, MO, USA) and stored at −20 °C. The conjugation was confirmed using ^1^H NMR using a Bruker Avance III HD 400 MHz NMR Spectrometer. Rhodamine conjugated dendrimers were surface modified to have either a neutral charge by acetylation or a negative charge by carboxylation reactions. For the acetylation process, dendrimers in methanol (10 mg) were reacted with acetic anhydride (2.17 × 10^−4^ mmol) in the presence of triethanolamine (1.25 molar excess of acetic anhydride) for 24 h at room temperature. For the carboxylation process, on the other hand, the dendrimers in DMSO were reacted with succinic anhydride (2.17 × 10^−4^ mol) for 24 h at room temperature. The surface-modified dendrimers were both purified by membrane dialysis, lyophilized, and characterized by ^1^H NMR. The surface charge of the dendrimers was measured using zeta potential with a Malvern Zetasizer Nano ZS (Malvern, UK).

### 2.4. Nanoparticle Tracking Analysis (NTA)

Diluted solutions of EVs were analyzed using an NS300 NTA instrument (Malvern Panalytical Ltd., Worcestershire, UK) with a 532 nm laser. Three videos that are at least 30 s each were collected for all samples and analyzed with the Nanosight 3.0 software. The particle concentration and size distribution of the EVs were calculated from three replicates.

### 2.5. Hypotonic Solution Preparation

The osmolality of the complete media and modified hypotonic media were measured using an Advanced Instruments osmometer at room temperature. Hypotonic buffer was composed of 125 mg/L monosodium phosphate, 3.7 g/L sodium bicarbonate, 4.5 g/L glucose, 1 mM sodium pyruvate, and 1% (*v*/*v*) penicillin/streptomycin. Hypotonic buffer was mixed with DMEM media supplemented with 1% (*v*/*v*) penicillin/streptomycin (335 mOsm/kg) at a ratio of 1:2, 1:1, or 2:1 to make a hypotonic media having osmolality of 253, 223, and 190 mOsm/kg, respectively. 

### 2.6. Assessment of Cytotoxicity of CB Treatment and Osmotic Pressure

A CCK-8 assay was utilized to measure the cell viability upon CB treatment (0, 5, 10, and 20 µg/mL) and/or osmotic pressure (335, 253, 223, and 190 mOsm/kg) treatment. Briefly, cells were cultured in 96-well plates until they reached 60–80% confluence. Cells were incubated in 100 µL serum-free media with CB and/or osmotic pressure for 24 h. CCK-8 reagent (10 µL) was added to each well and incubated at 37 °C for 2 h. The cell viability was determined by measuring absorbance at 450 nm using a plate reader. 

### 2.7. Protein Concentration Measurement

Total protein concentration of cells and EVs were measured from their lysates. To prepare lysates, EVs and cells were incubated with RIPA buffer for 30 min at 4 °C. The protein concentration was measured with BCA assay using the manufacturer’s instructions. 

### 2.8. Transmission Electron Microscopy (TEM) Imaging

The immunogold staining procedure was carried out to test for the presence of exosomal membrane proteins in hybrid NPs using antibodies against CD63 and tyrosine-protein kinase Met (C-Met). For immunogold staining, fixed hybrids NPs and exosomes were adsorbed onto 300 mesh Formvar/Carbon grid and blocked with 0.1% BSA for 30 min. The grids were then washed and incubated overnight at 4 °C with an antibody against CD63 (polyclonal anti-CD63 antibody, SC-15363, Santa Cruz Biotechnology, San Jose, CA, USA) and C-Met (polyclonal anti-c-Met antibody, AF276, R&D Systems, Minneapolis, MN, USA). All the grids were rinsed and floated on 10 nm gold conjugated secondary antibody (EM Goat anti-Rabbit or Rabbit anti-goat IgG: 10 nm Gold, BBI Solutions) for 1 h at room temperature. The grids were washed, fixed in 2% glutaraldehyde, and contrasted with 1% uranyl acetate solution. The grids were imaged with Tecnai T-12 TEM (FEI, Hillsboro, OR, USA) and Gatan Ultrascan CCD camera (Gatan, Pleasanton, CA, USA).

### 2.9. Western Blotting

The EVs were lysed using RIPA buffer and quantified for protein concentration using a BCA Assay, as described previously [28]. The proteins were resolved on an acrylamide gel and transferred onto PVDF membrane in wet transfer conditions. The blot was blocked with 5% skim milk for 1 h followed by overnight incubation in primary antibody against CD81 (monoclonal anti-CD81 SC-166028, Santa Cruz Biotechnology, San Jose, CA, USA) and macrophage migration inhibitory factor (MIF) (monoclonal anti-MIF, MAB289, R&D Systems) at 4 °C. Blots were incubated in secondary antibodies for 1 h at room temperature and washed. The blots were developed using a chemiluminescent reagent, Clarity Western ECL Substrate (Bio-Rad), and imaged using Syngene G:Box F3 (Syngene, Frederick, MD, USA).

### 2.10. Cell Uptake and Confocal Microscopy Imaging

ACHN cells were seeded in 8-well chamber slides (Nunc™ Lab-Tek™ II Chamber Slide™ System, Thermo Scientific, Waltham, MA, USA) at a density of 25,000 cells/well and incubated for 24 h. EVs were labeled with DiO dye by incubation at 37 °C for 30 min followed by ultracentrifugation to remove the free dye. Cells were treated with labeled EVs for 4 h, washed, and fixed with 4% paraformaldehyde. The fixed cells were washed and stained for the nuclei using 1 µg/mL solution of Hoechst dye. The stained cells were washed and mounted using Prolong Gold mounting media and cover glass. The slides were imaged using Zeiss LSM 710 confocal microscope (CLSM, Carl Zeiss, Jena, Germany). The laser lines 405 and 488 nm were used to excite Hoechst (nuclei), and DiO (EV).

### 2.11. Dendrimer/DOX Loading

EVs (~10^9^ particles as measured by NTA) were aliquoted in 500 µL of PBS and mixed with 1 µg of dendrimer or doxorubicin. The mixture was sonicated using a QSonica sonicator (20 kHz) with a 3.2 mm probe at 20% amplitude with 10 s of on and off pulses for a total of 3 pulses, followed by incubation at 37 °C for 30 min for the membranes to reform. The number of dendrimers or doxorubicin-loaded into the EVs was measured using fluorescence intensity measurements with a Synergy microplate reader (Biotek). The amount loaded was normalized to the number of EVs as measured by NTA.

### 2.12. Cell Viability Measurement

ACHN cells were seeded in a 96-well plate at a density of 12,000 cells/well and incubated for 24 h. The cytotoxicity of DOX-loaded EVs (DOX concentration of 1 or 2 µM) was measured using CCK-8 as described in Section 2.6. Cell viability of 100% was defined as the viability of control cells that were incubated simultaneously without addition of any EVs. Data were obtained from three independent experiments.

### 2.13. Statistical Analysis

The data obtained from this study were presented without any pre-processing. The statistical significance was analyzed using SPSS^®^ Statistics 26 (IBM Corp., Armonk, NY, USA). A two-tailed Student’s *t*-test was utilized to investigate the statistical difference between different groups unless noted otherwise (*n* ≥ 3).

## 3. Results and Discussion

### 3.1. Cytochalasin B Treatment Increases EV Secretion

**Figure 1:**
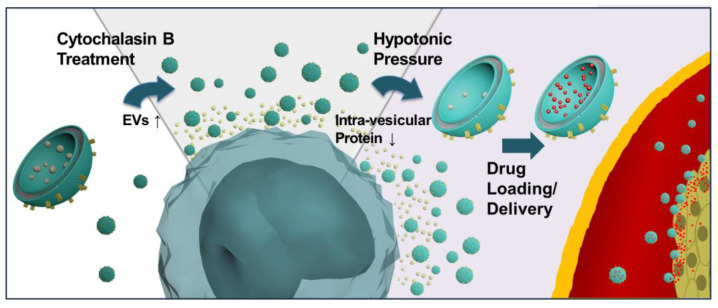
A schematic diagram illustrating the synergistic effect of cytochalasin B treatment and hypo-osmotic pressure for the up-scale of EV production and enhanced drug loading of the produced EVs.

Figure 1 demonstrates the design scheme of hypo-CIMVs. Two different renal cell carcinoma (RCC) cell lines, ACHN and 786-O, were used in this study. The cells were treated with CB under hypotonic conditions to increase the secretion of EVs and reduce the intravesicular content for the efficient loading and delivery of chemo-drugs. The CB treatment promotes the budding of vesicles by disorganizing the actin cytoskeleton, and this mechanism is known to have only a minor effect on cell viability to a certain extent [29]. However, CB induces cell shrinkage and nuclear condensation, which leads to cell apoptosis when it exceeds a certain threshold, producing EV-sized impurities such as apoptotic bodies [22,30]. Thus, we first determined the CB concentration that increases EV secretion with a minimal apoptotic body formation.

**Figure 2:**
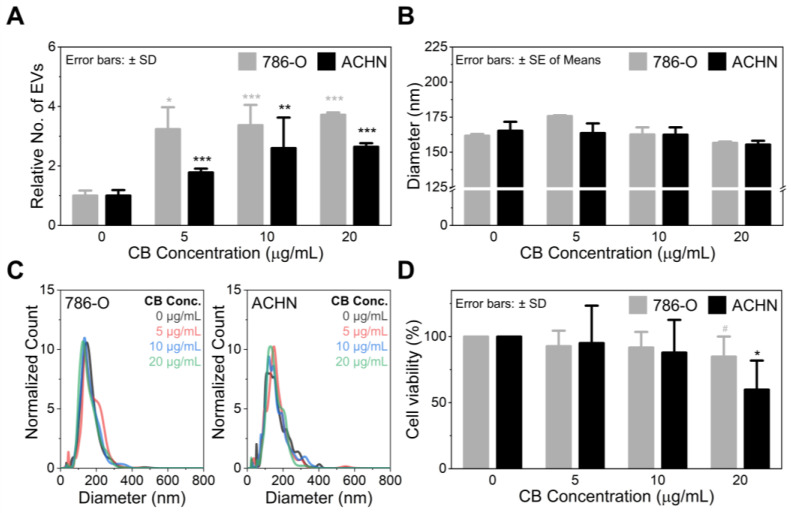
The effect of cytochalasin B on (**A**) EV production, (**B**,**C**) size distribution of the produced EVs, and (**D**) cell viability. Significance levels are indicated as # *p* < 0.10, * *p* < 0.05, ** *p* < 0.01, and *** *p* < 0.001, compared to the non-CB treated cells. NTA data were obtained from at least three independent experiments, performed in triplicates (*n* ≥ 3). The cell viability was measured from three independent experiments, performed in duplicates (*n* = 3).

As demonstrated in Figure 2A, CB significantly increased the EV secretion from 786-O cells by releasing 3.2-fold (*p* = 0.032), 3.4-fold (*p* < 0.001), and 3.7-fold (*p* < 0.001) more EVs than the naturally secreted EVs at the CB concentrations of 5, 10, and 20 µg/mL, respectively. Likewise, CB treatment increased the budding of EVs from ACHN cells, releasing 1.8-fold (*p* < 0.001), 2.8-fold (*p* = 0.009), and 2.6-fold (*p* < 0.001) more EVs at the given CB concentrations. The size of the CIMVs was within the range of naturally secreted EVs, regardless of CB concentration (Figure 2B,C).These results also agree with earlier studies that demonstrated CIMVs and naturally secreted EVs have a diameter of 100–1000 nm [21,22,31,32,33]. Note that the higher EV secretion from 786-O cells than ACHN cells is presumably due to their difference in proliferation rate, which is regulated by the actin cytoskeleton inside the cells [34]. CB disrupts the actin cytoskeleton generating EVs from cells, and its activity may be more prominent in rapidly proliferating cells (i.e., the 786-O cells used in this study). However, the exact mechanism needs further investigation. It should also be noted that CB promotes EV production not only from RCC cells but also from other cell types, including other types of cancer cells, mesenchymal stem cells, and immune cells, as described in Figure S1 and previous publications.

As previously mentioned, an increase in CB concentration disrupts the actin cytoskeleton, potentially leading to cell apoptosis [30]. CB-induced cell death is well-described in Figure 2D, which shows the inverse correlation between the viability of cancer cells and CB concentration. However, the cytotoxicity of CB was statistically insignificant until its concentration reached 10 µg/mL. It was found that cell viability abruptly decreased when CB concentration increased from 10 to 20 µg/mL. This effect was more pronounced in ACHN cells, which showed a cell viability reduction from ~88% to ~60% (*p* = 0.039) as the CB concentration increased from 10 to 20 µg/mL. The optimal CB concentration was thus determined to be 10 µg/mL, which increases the secretion of EVs by ~three-fold with only ~10% cell death.

### 3.2. Hypo-Osmotic Pressure Helps Release Intracellular Proteins without Affecting CB Activity

Extracellular osmolarity is known to regulate cell volume and shape [35]. Under hypotonic conditions, cells swell due to rapid water intake through the plasma membrane, followed by a slow recovery near their initial size (typically 20–100 min) by transporting ions and osmolytes to the extracellular domain [36,37,38,39,40,41]. An earlier study has shown that cancer cells can swell up to ~70% of their original volume in hypotonic conditions, only 2 min after stimulation at an osmolality of 190 mOsm/kg [38]. The cells then experience the regulatory volume decrease (RVD) that helps protect them from being lysed by slowly recovering to their original size [38]. We therefore hypothesized that cells would release a large amount of biological content (i.e., proteins, analytes, and nucleic acids) to the extracellular domain during the osmotic pressure-induced swelling. These cells would then, in turn, produce EVs which have a lower amount of intravesicular content and exhibit a higher capacity for drug loading than the naturally secreted EVs.

**Figure 3:**
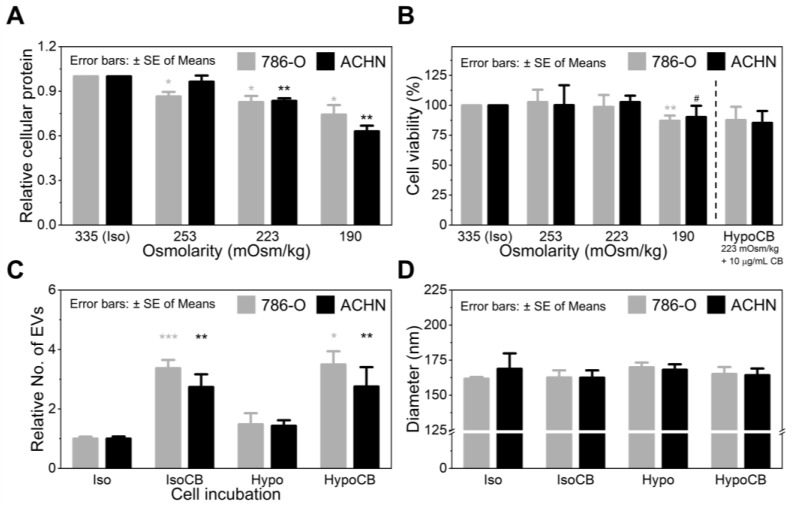
The effect of hypo-osmotic pressure on (**A**) amount of total cellular protein (*n* = 8), (**B**) cell viability (*n* = 4), (**C**) EV production (*n* ≥ 3), and (**D**) size of the produced EVs (*n* ≥ 3). Significance levels are indicated as # *p* < 0.10, * *p* < 0.05, ** *p* < 0.01, and *** *p* < 0.001, compared to the non-CB treated cells.

To verify our hypothesis, we first investigated the changes in intracellular protein levels depending on the osmolality of the cell culture media. As demonstrated in Figure 3A, the amount of cellular protein was proportional to the osmolality of the cell culture media; the amount of cellular protein decreased as the osmolality decreased. In particular, compared to the isotonic condition (335 mOsm/kg), 24 h incubation at 223 mOsm/kg resulted in ~17% less cellular protein for both 786-O (*p* = 0.025) and ACHN (*p* = 0.003) cells, with no significant cytotoxic effect (*p* > 0.403) (Figure 3B). The decrease in osmolality to 190 mOsm/kg further reduced the amount of cellular protein (25–37% reduced) but the cell viability was also heavily affected at the given osmolarity, with ~10% (*p* = 0.060) and ~13% (*p* = 0.009) viability loss from ACHN and 786-O cells, respectively. Therefore, we set the optimal hypo-osmotic pressure to be 223 mOsm/kg, at which the osmotic pressure forces cells to release their intracellular content to the extracellular domain without any noticeable cytotoxic effect.

Next, we explored whether hypo-osmotic pressure (223 mOsm/kg) can affect EV secretion of cancer cells. As demonstrated in Figure 3C, there was no significant difference in EV secretion between the cells cultured in isotonic and hypotonic media. Furthermore, hypo-osmotic pressure did not affect the activity of CB (10 µg/mL), as the number of EVs released by CB was independent of the osmolality of the culture media. Specifically, under hypotonic conditions, CB treatment produced 3.5-fold (786-O; *p* = 0.028) and 2.8-fold (ACHN; *p* = 0.005) more EVs than the non-treated cells. This difference was equivalent to the increase in EV secretion due to the CB treatment alone, without hypo-osmotic pressure (3.4-fold enhanced for 786-O and 2.8-fold enhanced for ACHN cells).

NTA assay further demonstrated that osmotic pressure did not affect the size of the produced EVs (Figure 3D). The average mean size of the produced EVs was between 160–170 nm, regardless of osmolality and/or CB treatment (Figure S2). Another important result we found is that hypo-osmotic pressure of 223 mOsm/kg did not induce further apoptosis of CB-treated cells (Figure 2D,B). The difference in the viability of the CB-treated cells in isotonic and hypotonic conditions was statistically insignificant (*p* = 0.635 for 786-O and *p* = 0.848 for ACHN). Altogether, these results support that the given osmotic pressure neither affects CB activity nor cells’ EV secretion. 

### 3.3. CB Treatment and Hypo-Omotic Pressure Synergistically Enhances Loading Capacity 

**Figure 4:**
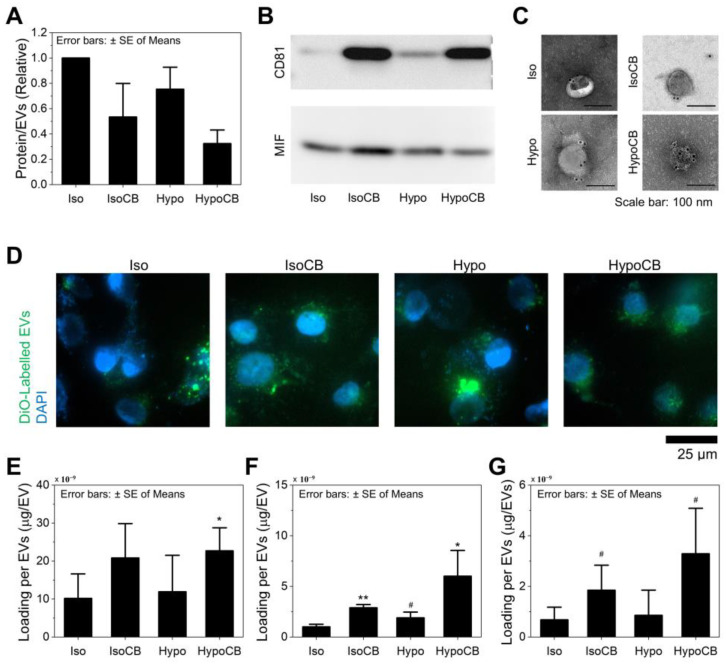
The proteins expressions, cellular uptake, and dendrimer loading capability of hypo-CIMVs compared to naturally-secreted EVs, hypo-EVs, and CIMVs: (**A**) Amount of total vesicular protein (*n* = 3); (**B**) Expression of transmembrane protein CD81 compared to intravesicular protein MIF; (**C**) Immunogold TEM images for assessing the CD63 (transmembrane marker) expression; (**D**) Cellular uptake of the produced EVs; (**E**–**G**) Loading of (**E**) G7-NH2, (**F**) G7-COOH, and (**G**) G7-Ac to the produced EVs. Significance levels are indicated as # *p* < 0.10, * *p* < 0.05, and ** *p* < 0.01, compared to the EVs obtained from non-CB treated cells. The dendrimer loading to the EVs was analyzed from at least three independent experiments (*n* ≥ 3).

We assessed the protein-to-particle ratio of EVs obtained from ACHN cells at various incubation conditions: naïve EVs, hypo-EVs (EVs secreted from the cells under hypotonic conditions), CIMVs (EVs released from the cells via CB treatment), and hypo-CIMVs (EVs released from the cells via CB treatment at the hypotonic conditions). The hypo-EVs had ~25% (*p* = 0.066) less total vesicular protein than the same number of naïve EVs (Figure 4A), supporting our hypothesis that EVs derived from cells with less intracellular protein would also exhibit less vesicular protein. Interestingly, CB treatment also reduced total vesicular protein, which was ~47% (*p* = 0.039) less than protein obtained from the same number of naïve EVs. This again agrees with the previous study that investigated the effect of CB on EV secretion, which showed increased EV numbers in comparison to total protein content. We, therefore, assumed that the speed of EV formation accelerated the synthesis of intravesicular materials, inducing cells to secrete the EVs with a lower amount of vesicular protein. Resultingly, the combination of hypo-osmotic pressure and CB treatment synergistically reduced the amount of vesicular protein by ~68% (*p* = 0.001). 

A western blot analysis was then performed to examine the expression profiles of two human proteins, CD81 and MIF, which are expressed on the transmembrane and cytoplasm, respectively [42,43]. Note that an equal amount of protein was loaded across immunoblot lanes for all four EV groups. As shown in Figure 4B and Figure S3, the CD81-to-MIF ratio (membrane protein vs. cytoplasm protein) was the highest for hypo-CIMVs, whereas naïve EVs showed the lowest CD81 expression. More specifically, the CD81-to-MIF ratio was inversely proportional to the protein-per-EV ratio which was previously determined in this study (Figure 4A). Considering that the surfaces of all four EV groups are formed from the lipid bilayers obtained from the same cell line (ACHN), the difference in the total amount of protein between each EV type is presumably due to the loss of intravesicular proteins by hypotonic pressure and CB treatment. Immunogold transmission electron microscopy (TEM) further demonstrated that all four types of EVs express CD63, which is one of the most well-established EV transmembrane markers, as well as C-Met, which is overexpressed on the surface of ACHN cells (Figure 4C and Figure S4) [26,44]. We also found that all four EVs had similar levels of charge density on their surfaces (Figure S5). As a result, these EVs exhibited a similar level of cellular uptake from ACHN cells (Figure 4D), implying that neither CB treatment nor hypo-osmotic pressure results in a noticeable effect on the expression levels of transmembrane proteins that play a critical role in determining the route and amount of EVs’ cellular uptake. 

We then examined the drug loading efficiencies of the produced EVs and investigated the correlation with the amount of intravesicular protein. Generation 7 (G7) polyamidoamine (PAMAM) dendrimers were employed as a model drug due to their high degree of chemical versatility for manipulating the surface charge density [45,46,47,48]. Three types of G7 dendrimers, having either positive (G7-NH_2_), neutral (G7-Ac), or negative (G7-COOH) charges were prepared using our previously developed protocols. ^1^H NMR spectroscopy and zeta potential analyzer confirmed the presence of each periphery end group with a zeta potential of 55.9 ± 2.8, 1.4 ± 0.5, and −27.9 ± 0.9 mV for G7-NH_2_, G7-Ac, and G7-COOH, respectively (Figures S6 and S7). However, regardless of the surface charge of the dendrimers, the hypo-CIMVs demonstrated the highest loading efficiency amongst the four EV groups (Figure 4E–G). Specifically, for G7-Ac and G7-COOH, 4.8-fold (*p* = 0.059) and 6.0-fold (*p* = 0.027) more dendrimers were loaded on the hypo-CIMVs compared to the naïve EVs, which was remarkably greater than the other three groups. The hypo-CIMVs also displayed the highest loading efficiency for the G7-NH_2_, but the difference was less remarkable (2.2-fold increased; *p* = 0.035) due to the non-specific adsorption of the positively charged dendrimers on vesicle surfaces. The hypo-EVs or CIMVs also exhibited higher loading of dendrimers than the naïve EVs, but the differences were less remarkable than that of hypo-CIMVs. Collectively, these findings suggest that the combination of hypo-osmotic pressure and CB treatment enables the large-scale production of EVs with less intravesicular content, allowing for the EVs to enclose a larger cargo into their cytoplasm than naturally secreted EVs.

### 3.4. The Combination of CB Treatment and Hypo-Osmotic-Pressure-Improved Drug Delivery

**Figure 5:**
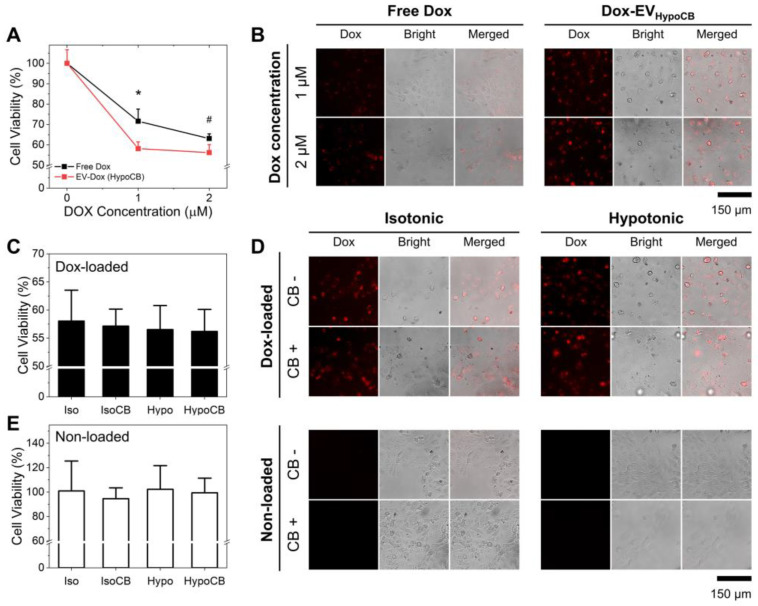
DOX delivery efficiency of the produced EVs: (**A**,**B**) The in vitro cytotoxic efficacy of the DOX-loaded hypo-CIMVs compared to the free DOX; (**C**,**D**) The in vitro cytotoxic efficacy of the DOX-loaded hypo-CIMVs compared to the DOX loaded in the naturally secreted EVs, CIMVs, and hypo-EVs at the DOX concentration of 2 µM; (**E**) The cytotoxicity of the EVs without DOX loading. Note that the cell viability of 100% corresponds to the viability of cells that were incubated simultaneously without addition of any EVs. Data were presented as mean ± standard deviation of three experiments (*n* = 3). Significance levels are indicated as # *p* < 0.10 and * *p* < 0.05, compared to EVs obtained from the non-CB treated cells.

The therapeutic potential of hypo-CIMVs as a nanocarrier for the delivery of chemo-drugs was investigated by loading doxorubicin (DOX) onto the EVs. As demonstrated in Figure S8, the hypo-CIMVs achieved the highest DOX loading compared to the other three EV groups—3.9-fold (*p* = 0.022) higher than naïve EVs. We compared the DOX delivery efficiency of hypo-CIMVs with free DOX by performing a CCK8 assay after 6 h incubation of ACHN cells with either free DOX or the DOX-loaded hypo-CIMVs at a DOX concentration of 1 or 2 µM. Note that for the hypo-CIMVs, the free DOX was removed via ultracentrifugation and the DOX concentration between the two groups was balanced based on the fluorescent intensity of DOX. As demonstrated in Figure 5A,B, the loading of DOX to the hypo-CIMVs increased the delivery of the therapeutic cargos to the cells. Specifically, the treatment of DOX-loaded hypo-CIMVs on ACHN cells resulted in 41.8 ± 3.3% and 43.8 ± 3.9% viability reduction at the DOX concentration of 1 and 2 µM, respectively, which was more effective than the free DOX that killed 28.4 ± 6.0% (*p* = 0.027) and 36.9 ± 2.3% (*p* = 0.058) at the given DOX concentrations. 

The DOX delivery efficiency of the hypo-CIMVs was then compared with the other EVs used in this study. As demonstrated in Figure 5C,D, all four types of DOX-loaded EVs had exhibited a similar level of cancer cytotoxicity. Specifically, the viability of ACHN cells were 58.0 ± 5.5%, 57.1 ± 3.3%, 56.5 ± 3.7%, and 56.2 ± 3.9% when treated with naïve EVs, hypo EVs, CIMVs, and hypo-CIMVs, respectively, which were loaded with 2 µM DOX. This was in good agreement with the cellular uptake analysis (Figure 4C), which revealed that all four EVs have a similar level of internalization into ACHN cells. Considering that the hypo-CIMVs exhibited the highest loading efficiency among the EV groups, hypo-CIMVs can achieve equivalent therapeutic and pharmacological effect compared to naturally secreted EVs, even with a smaller dose. It should be note that the internalization of bare EVs (without DOX) did not have any noticeable cytotoxic effect on ACHN cells (Figure 5E).

## 4. Conclusions

EVs have demonstrated several advantages over other currently available synthetic drug delivery vehicles, including high stability, low cytotoxicity, and intrinsic cell targeting properties. However, low yield and drug loading capacity limited the clinical translation of EV-based drug delivery systems. In this study, we developed an engineering strategy that facilitates the upscaled production of EVs and improves their drug loading capacity by promoting EV secretion via CB treatment and reducing intravesicular contents via hypo-osmotic pressure. CB treatment (10 µg/mL) induces the budding of EVs from the cell membrane, increasing cells’ EV secretion by ~three-fold compared to the number of naturally secreted EVs. The addition of hypo-osmotic pressure (223 mOsm/kg) to CB treatment promoted cells releasing their intracellular contents to the extracellular domain, producing EVs with less intravesicular contents. As a result, the produced hypo-CIMVs (EVs released from the cells via CB treatment at hypotonic conditions) were capable of loading 3.4-fold more DOX than the same number of naturally secreted EVs. The DOX-loaded hypo-CIMVs were then utilized for in vitro cytotoxicity assay, and demonstrated a 50% increased therapeutic effect compared to the same amount of free DOX (1 µM). Obviously, hypo-CIMVs need to be further tested using more in vitro and in vivo assays to confirm our new protocol’s therapeutic efficacy and safety. Specifically, extensive in vivo studies such as tumor accumulation, serum stability, cytotoxicity, therapeutic efficacy, and biodistribution will be the subject of our next publication. In summary, the results presented herein reveal that the combination of CB treatment and hypo-osmotic pressure are capable of efficiently upscaling EV production and drug loading capacity, and therefore have the potential to be utilized as novel nano-vehicles for the delivery of chemotherapeutic agents.

## Data Availability

The data presented in this study are available on request from the corresponding author.

## Supplementary Materials

The following supporting information can be downloaded at: https://www.mdpi.com/article/10.3390/nano12010003/s1. Figure S1: EVs obtained from MCF-7 cells at CB concentrations of 0 and 10 µg/mL, Figure S2: Size distribution of the produced EVs, which include naïve EVs (grey), CIMVs (yellow), hypo-EVs (cyan), and hypo-CIMVs (pink), Figure S3: The whole western blot membrane images, Figure S4: Immunogold TEM images for assessing CD63 and C-Met expressions on EVs, Figure S5: Zeta potential measurements of the EVs produced at different cell incubation conditions, Figure S6: 1H NMR spectra of surface-modified G7 PAMAM dendrimers, Figure S7: Zeta potential measurements of surface-modified G7 PAMAM dendrimers, Figure S8: Relative DOX-loading efficiency of the EVs produced at different cell incubation conditions. Significance levels are indicated as # *p* < 0.10, * *p* < 0.05, ** *p* < 0.01, and *** *p* < 0.001, compared to the EVs obtained from non-CB treated cells.
