# Supplementary material for: Cytochalasin B Treatment and Osmotic Pressure Enhance the Production of Extracellular Vesicles (EVs) with Improved Drug Loading Capacity
Source: Nanomaterials (Basel). 2021 Dec 21;12(1):3. doi: 10.3390/nano12010003 (PMC8746776; doi:10.3390/nano12010003)
Supplement: Supplementary file 1 [file nanomaterials-12-00003-s001.zip › nanomaterials-1465132-supplementary.pdf]

nanomaterials

MDPI

Supplementary Materials

Cytochalasin B Treatment and Osmotic Pressure Enhance the Production of Extracellular Vesicles (EVs) with Improved Drug Loading Capacity

Ashita Nair [1,2,†], Jiyoon Bu [1,2,†], Piper A. Rawding [1,2], Steven C. Do [1], Hangpeng Li [1] and Seungpyo Hong [1,2,3,4,*]

[1] Division of Pharmaceutical Sciences, School of Pharmacy, University of Wisconsin, Madison, WI 53705, USA; anair23@wisc.edu (A.N.); bu6@wisc.edu (J.B.); rawding@wisc.edu (P.A.R.); scdo@wisc.edu (S.C.D.); hli578@wisc.edu (H.L.)
[2] Wisconsin Center for NanoBioSystems (WisCNano), School of Pharmacy, The University of Wisconsin-Madison, 777 Highland Ave., Madison, WI 53705, USA
[3] Department of Biomedical Engineering, College of Engineering, University of Wisconsin, Madison, WI 53706, USA
[4] Yonsei Frontier Lab, Department of Pharmacy, Yonsei University, Seoul 03722, Korea
* Correspondence: seungpyo.hong@wisc.edu
† A Nair and J Bu contributed equally to this work.

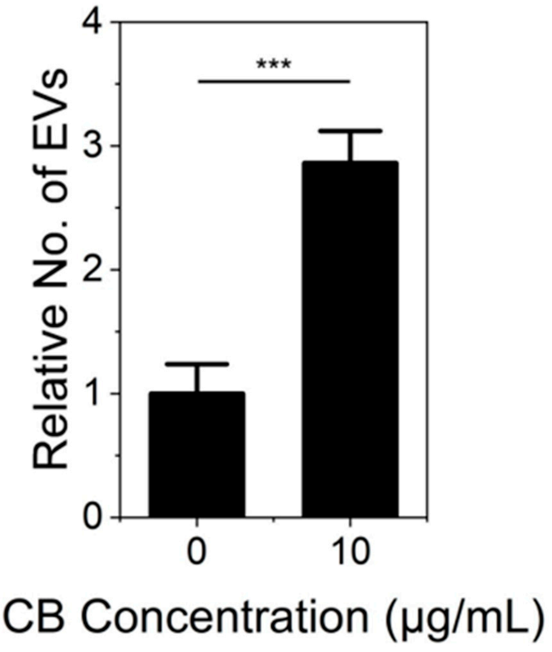

Figure S1. EVs obtained from MCF-7 cells at CB concentrations of 0 and 10 μg/mL. ***p <0.001.

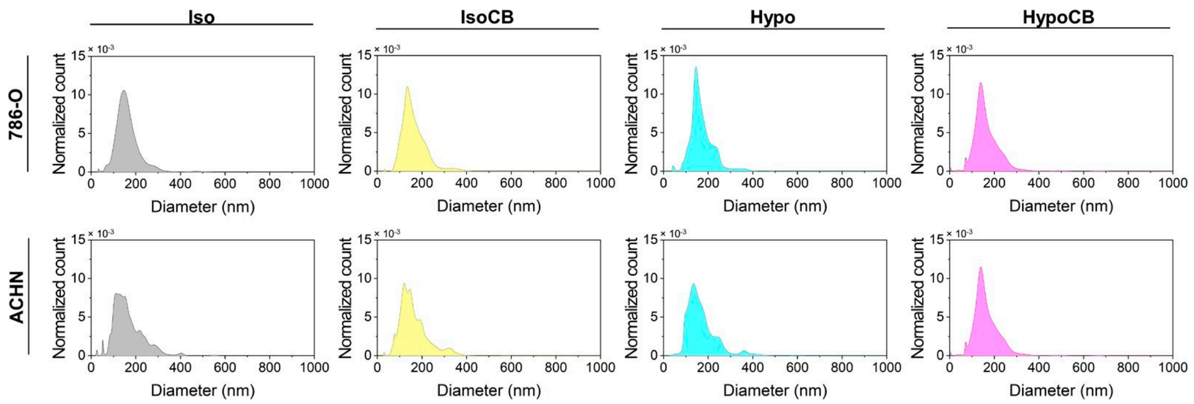

Figure S2. Size distribution of the produced EVs, which include naïve EVs (grey), CIMVs (yellow), Hypo-EVs (cyan), and Hypo-CIMVs (pink).

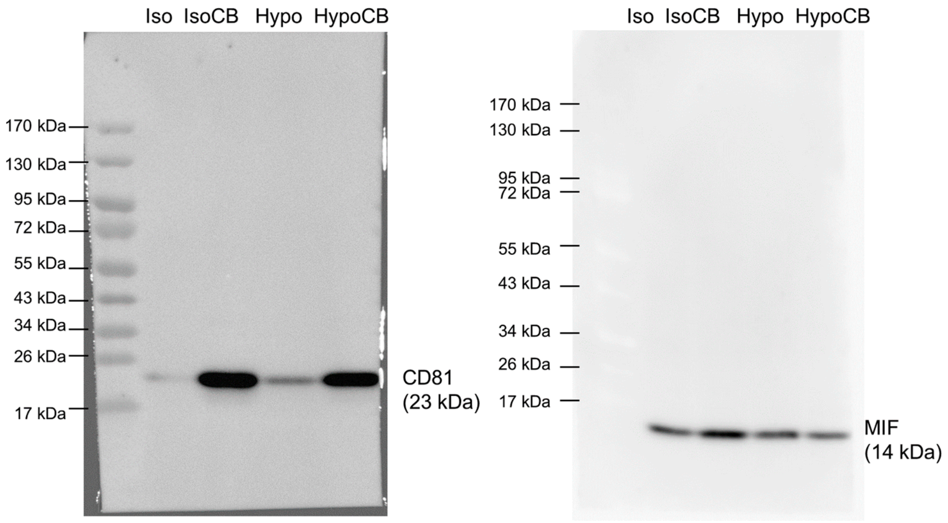

Figure S3. The whole Western Blot membrane images.

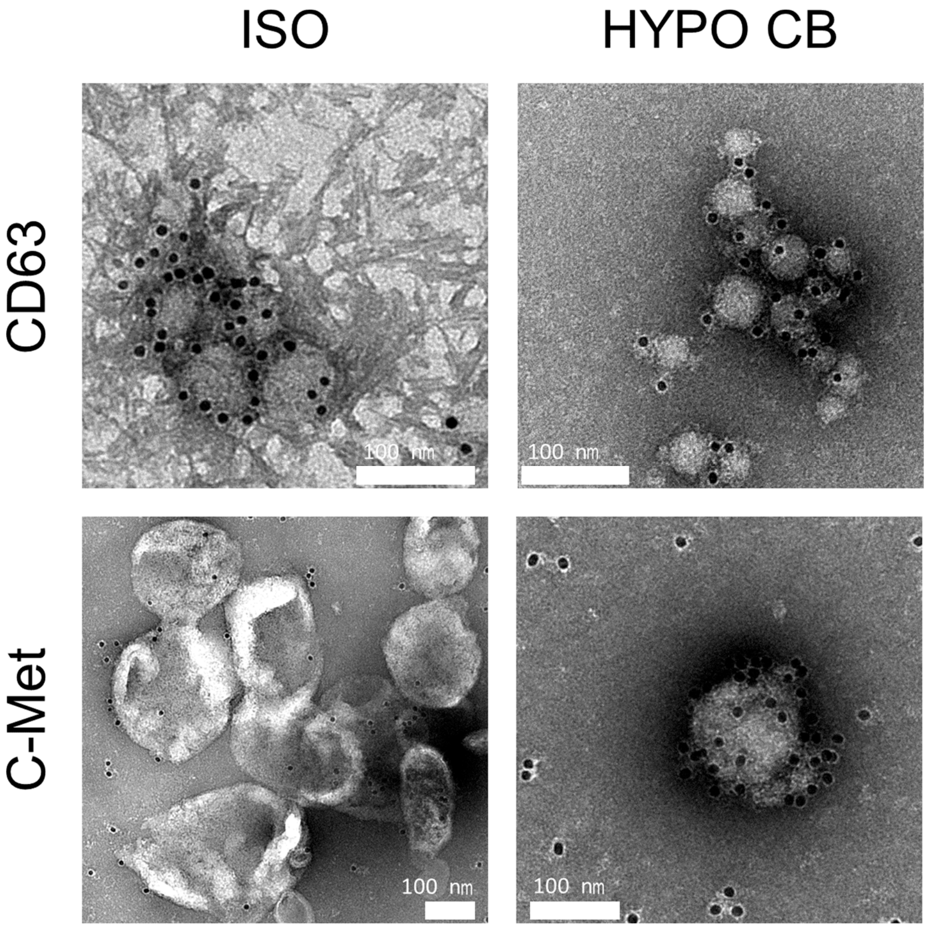

Figure S4. Immunogold TEM images for assessing CD63 and C-Met expressions on EVs.

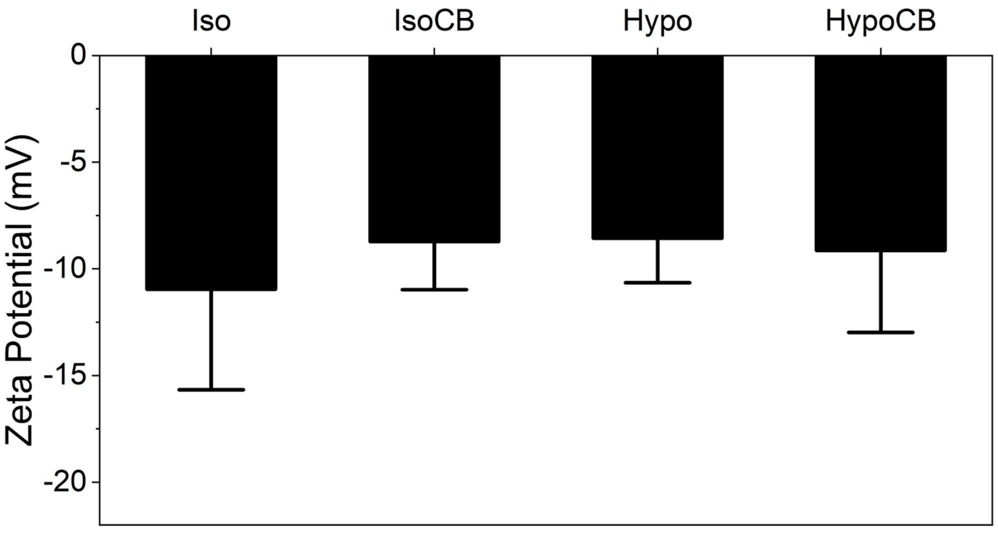

Figure S5. Zeta potential measurements of the EVs produced at different cell incubation conditions.

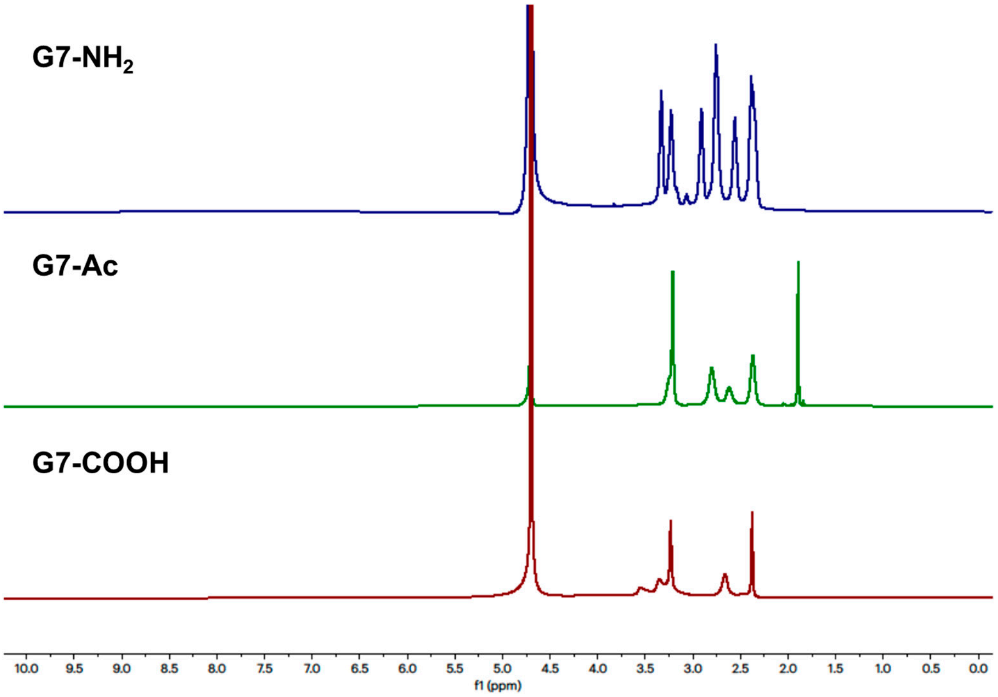

Figure S6. ^{1}H NMR spectra of surface-modified G7 PAMAM dendrimers.

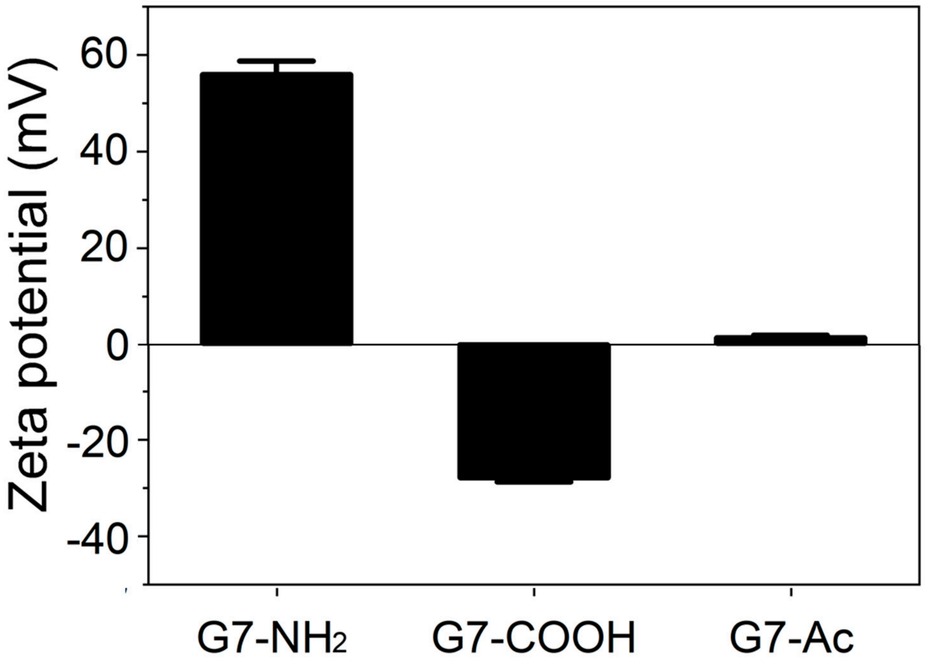

Figure S7. Zeta potential measurements of surface-modified G7 PAMAM dendrimers.

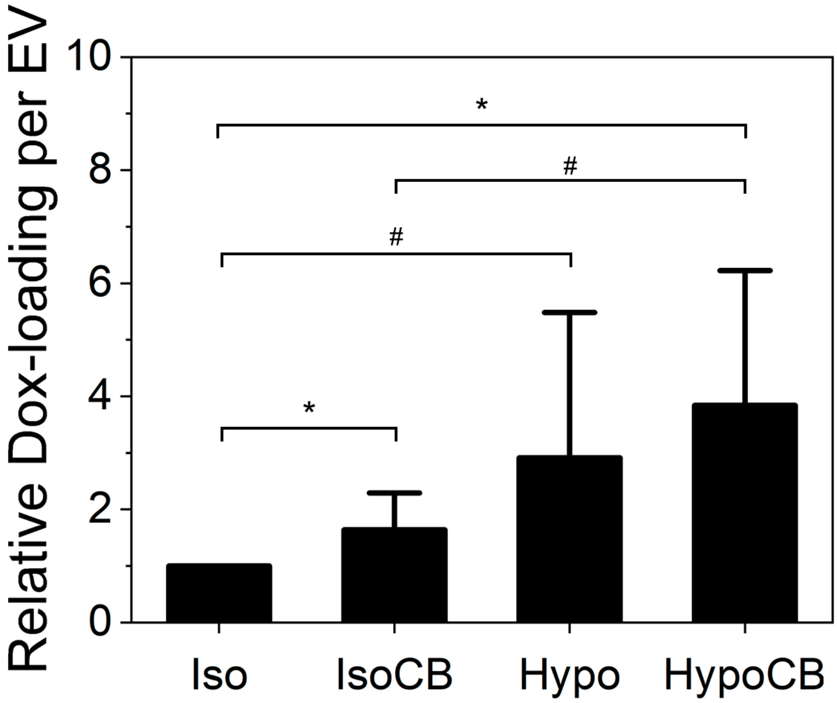

Figure S8. Relative Dox-loading efficiency of the EVs produced at different cell incubation conditions. Significance levels are indicated as #p <0.10, *p <0.05, compared to the EVs obtained from non-CB treated cells.
